# Smoking and Elevated Preneoadjuvant Chemoradiotherapy Serum Carcinoembryonic Antigen Levels Are Associated With High Tumor Regression Grade and Poor Survival in Patients With Locally Advanced Rectal Cancer

**DOI:** 10.1002/kjm2.70008

**Published:** 2025-03-13

**Authors:** Jen‐Pin Chuang, Hsiang‐Lin Tsai, Po‐Jung Chen, Ching‐Wen Huang, Wei‐Chih Su, Tsung‐Kun Chang, Yen‐Cheng Chen, Ching‐Chun Li, Yung‐Sung Yeh, Jaw‐Yuan Wang

**Affiliations:** ^1^ Department of Surgery Chiayi Hospital, Ministry of Health and Welfare Chiayi City Taiwan; ^2^ Department of Surgery, Faculty of Medicine, College of Medicine National Cheng Kung University Tainan Taiwan; ^3^ Department of Surgery National Cheng Kung University Hospital Tainan Taiwan; ^4^ Division of Colorectal Surgery, Department of Surgery Kaohsiung Medical University Hospital, Kaohsiung Medical University Kaohsiung Taiwan; ^5^ Department of Surgery, Faculty of Medicine, College of Medicine Kaohsiung Medical University Kaohsiung Taiwan; ^6^ Graduate Institute of Clinical Medicine, College of Medicine Kaohsiung Medical University Kaohsiung Taiwan; ^7^ Department of Surgery, Faculty of Post‐Baccalaureate Medicine, College of Medicine Kaohsiung Medical University Kaohsiung Taiwan; ^8^ Division of Colorectal Surgery, Department of Surgery Kaohsiung Municipal Hsiaokang Hospital Kaohsiung Taiwan; ^9^ Division of Trauma and Surgical Critical Care, Department of Surgery Kaohsiung Medical University Hospital, Kaohsiung Medical University Kaohsiung Taiwan; ^10^ Department of Emergency Medicine, Faculty of Post‐Baccalaureate Medicine, College of Medicine Kaohsiung Medical University Kaohsiung Taiwan; ^11^ Graduate Institute of Medicine, College of Medicine Kaohsiung Medical University Kaohsiung Taiwan; ^12^ Center for Cancer Research Kaohsiung Medical University Kaohsiung Taiwan

**Keywords:** carcinoembryonic antigen, neoadjuvant chemoradiotherapy, rectal cancer, smoking, tumor regression grade

## Abstract

Neoadjuvant chemoradiotherapy (NACRT) is the standard treatment for patients with locally advanced rectal cancer (LARC). Tumor regression grade (TRG) is an essential prognostic factor in determining treatment efficacy. However, the potential factors influencing TRG in patients with rectal cancer who have received NACRT have not been investigated. We conducted a retrospective analysis of patients with LARC who received NACRT followed by surgical resection. We collected data on the patient characteristics, including age, sex, comorbidities, tumor size, lymph node status, time between NACRT and surgery, and pretreatment carcinoembryonic antigen (CEA) levels. TRG was determined on the basis of a pathological assessment of resected specimens, and overall survival (OS) at 5 years was determined. Univariate and multivariate logistic regression models were employed to evaluate the association between the patient characteristics and TRG. Univariate analysis revealed that smoking and prechemoradiotherapy (pre‐CRT) and preoperative CEA levels were significantly associated with TRG. In a multivariate analysis, both smoking and higher pre‐CRT CEA levels were identified as significant predictors of a high TRG. The hazard ratios were 2.32 (95% confidence interval [CI]: 1.06–5.07, *p* = 0.036) for smoking and 3.1 (95% CI: 1.69–5.68, *p* < 0.001) for higher pre‐CRT CEA levels. In Kaplan–Meier analysis, the nonsmoker group exhibited higher OS (*p* = 0.004). Elevated pre‐CRT CEA levels and current smoking status were associated with a more than two fold increase in the risk of a higher TRG after NACRT. Moreover, smoking was a significant risk factor for poor OS in patients with LARC following NACRT.

## 1 Introduction

Colorectal cancer (CRC) is the second most common cause of cancer‐related death globally. The World Health Organization (WHO)'s Global Cancer Observatory report for 2020 revealed that over 1.9 million new cases of CRC were reported worldwide in that year, with rectal cancer accounting for approximately 732,210 of the new diagnoses [1]. Of these cases of rectal cancer, 5% to 10% were cases of locally advanced rectal cancer (LARC), which are graded as either stage II (cT3–T4, N0) or stage III disease (cT1–T4, N1–N3) [2, 3]. Primary LARC has historically been challenging to treat using surgery alone. The standard treatment for patients with stage II or III carcinoma of the rectum is preoperative neoadjuvant chemoradiotherapy (NACRT) followed by radical resection [4, 5, 6, 7]. Complete tumor response after neoadjuvant therapy for rectal cancer is associated with higher survival rates [8, 9]. Tumor regression grade (TRG) is used to categorize the histopathological responses of the primary tumor to chemoradiation and is useful for prognostication in these patients [5, 10]. The seventh edition of the *American Joint Committee on Cancer Staging Manual* recommends the use of TRG [11, 12].

Several TRG systems have been developed for evaluating the pathologic response of patients with rectal cancer to NACRT. Mandard et al. introduced a five‐category TRG calculated on the basis of the ratio of residual tumor cells to inflammatory fibrosis and was reported to be effective for assessing LARC tumor responses to NACRT [13]. The Dowrak–Rödel system, which also comprises five categories, assigns TRG numerically in the opposite direction to that of the Mandard system [5, 14]. The Mandard and Dowrak–Rödel systems were combined to form a three‐category system (TRG0 + 1, TRG2 + 3, TRG4) that was as effective as the original five‐category systems are [11]. The American Joint Committee on Cancer (AJCC) and the College of American Pathologists proposed a modified version of the Mandard system comprising four categories based on residual‐tumor‐cell scores [12, 15, 16]. This modified version enabled more accurate classification of the responses of patients with rectal cancer to NACRT [11, 15]. Apart from these four primary systems for evaluating response after NACRT, there are 14 other classification systems that include various modifications of TRGs [17].

Although TRG has been established as a significant predictor of disease‐free survival [10] and oncological outcomes in patients with rectal cancer following NACRT [5], little research has been conducted regarding the clinicopathological factors associated with TRG in such patients. Therefore, we conducted a comprehensive study to assess the effects of patient and tumor characteristics, the time between NACRT and surgery, and pretreatment serum carcinoembryonic antigen (CEA) levels on the development of TRG after chemoradiotherapy (CRT).

## 2 Materials and Methods

### 2.1 Patients

We identified 641 individuals who were given a diagnosis of LARC between 2014 and 2022. We considered patients who met the following criteria to be eligible for inclusion in the study: having received a preoperative pathological diagnosis of invasive rectal adenocarcinoma, having complete clinical and postoperative pathological data, and having undergone radical proctectomy after NACRT. Patients were excluded if they had stage I or IV rectal malignancy diagnoses, had received CRT for other neoplasms within 6 months before rectal cancer surgery, or had received a rectal cancer diagnosis at or before the age of 19 years.

Before undergoing treatment, the patients completed a series of pretreatment assessments, including physical examination, a medical history review, colonoscopy, tumor biopsy, chest radiography, abdominal computed tomography (CT), pelvic magnetic resonance imaging, a serum CEA assessment, and routine laboratory analysis. The clinical records of the patients were used to classify their clinical TNM tumor stages in accordance with the guidelines of the seventh edition of the *AJCC Cancer Staging Manual*. Approval for this study was obtained from the Institutional Review Board of Kaohsiung Medical University Hospital (approval number: KMUH‐IRB‐E(I)‐20210041).

### 2.2 Preoperative Therapy

Patients with T3, T4, or lymph node‐positive rectal cancer diagnoses were included in the study and had received preoperative NACRT in accordance with an established protocol [18]. Radiotherapy was administered at a dose of 45 Gy in 25 fractions to the entire pelvis. The patients subsequently received a 5.4‐Gy boost delivered in three fractions targeting the primary tumor. Concurrent chemotherapy was administered, with the chemotherapy involving a biweekly regimen of modified FOLFOX6 (mFOLFOX6) with radiotherapy. Each mFOLFOX6 cycle comprised oxaliplatin (85 mg/m^2^, on Day 1 only), folinic acid (400 mg/m^2^), and a 46‐h infusion of fluorouracil (2800 mg/m^2^). The patients underwent standard total mesorectal excision within 10–12 weeks after the completion of radiotherapy in accordance with our previously described protocol [18, 19].

### 2.3 Evaluation and Follow‐Up

The patients' post‐CRT responses were evaluated at approximately 6–10 weeks after CRT completion. Several clinical assessments were performed, including digital rectal examinations, colonoscopy, serum CEA tests, abdominal and chest CT scans, and pelvic magnetic resonance imaging. The post‐CRT locoregional stage was assessed using magnetic resonance imaging, and CT was used to evaluate distant metastasis. A cutoff value for preoperative CEA levels of ≤ 2 ng/mL was determined on the basis of prior research and was considered to be an independent clinical parameter for predicting pathological complete response following CRT in patients with LARC [20, 21]. After surgery, the patients made regular follow‐up visits to the outpatient department. The visits occurred every 3 months for the first 2 years and once every 6 months after that. Tumor recurrence within the pelvic region was classified as local failure, and recurrence outside the pelvis was considered distant failure. In line with our institution's guidelines, patients with stage II rectal cancer with high‐risk features or stage III LARC are recommended to receive adjuvant therapy after NACRT and subsequent radical surgery. Specifically, if postoperative pathology reveals a positive primary tumor (ypT+) or positive lymph nodes (ypN+), a total of 12 cycles of the FOLFOX regimen, including any cycles administered before surgery, are advised. On the other hand, patients who achieve a complete pathological response post‐surgery are recommended fluoropyrimidine‐based chemotherapy for up to 3 months after the operation [22].

### 2.4 Pathological Examination

**FIGURE 1:**
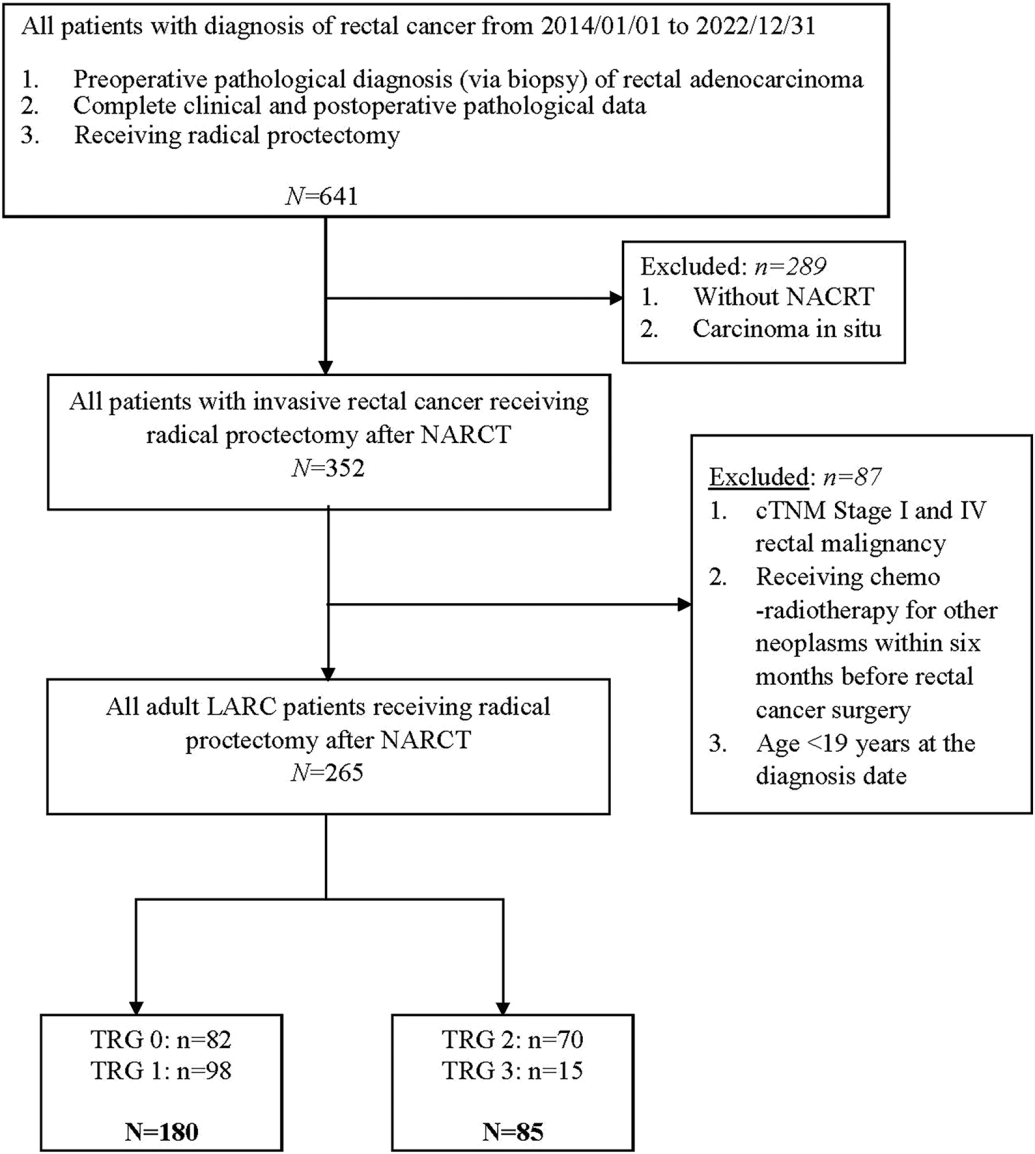
Protocol for patient inclusion.

Each tumor in the study population was classified on the basis of the WHO criteria [23] and was initially staged using the AJCC TNM system [12]. Resected rectal tumors were embedded in full thickness to enable evaluation of the circumferential resection margin and to assess whether the mesorectal excision was total. Sections from the rectal wall, which often included regional lymph nodes and perirectal tumor deposits, were examined. Two pathologists independently reviewed all tumors. The TRGs for the primary tumors and regional lymph nodes were determined using the following AJCC TRG system [12]: TRG 0 indicated the absence of viable cancer cells, TRG 1 indicated the presence of single or small groups of tumor cells (moderate response), TRG 2 indicated residual cancer surrounded by fibrosis (minimal response), and TRG 3 indicated minimal or no tumor cell destruction (poor response). The distribution of patients by TRG score is presented in Figure 1.

### 2.5 Statistical Analysis

Descriptive statistics were used to present the frequencies of categorical variables. A chi‐square test was performed to compare categorical data. Logistic regression was employed for univariate and multivariate analyses to identify factors associated with TRG. Hazard ratios (HRs) with 95% confidence intervals (CIs) were calculated to assess the risk of a poor response to NACRT. The Kaplan–Meier method was applied to determine the crude overall survival (OS) rate, and a log‐rank test was conducted to compare the distribution of time‐to‐event outcomes. A *p* value of < 0.05 was considered to indicate significance, and all *p* values were two‐tailed. The statistical analyses were performed using SPSS version 21.0 (IBM, Armonk, NY, USA).

## 3 Results

### 3.1 Patient Characteristics

A total of 265 eligible patients were included in the study (Figure 1). Of these patients, 180 were assigned to the TRG 0/1 group, and 85 were assigned to the TRG 2/3 group (Figure 1). The clinicopathological characteristics of the groups are listed in Table 1. The mean age was 62.2 ± 11.2 years, and 105 patients (39.6%) were women. Most of the study population had been given a diagnosis of cT3 (82.3%; 218/265) and were node positive (79.6%; 211/265). The proportion of smoking patients was higher in the TRG 2/3 group than in the TRG 0/1 group (25.9% vs. 12.8%; Table 1).

**TABLE 1:** Clinical and pathological characteristics of all eligible patients (*N* = 265).

	All eligible patients (*N* = 265)	
	TRG 0/1 (*n* = 180)	TRG 2/3 (*n* = 85)
Gender		
Male	107 (59.4%)	53 (62.4%)
Female	73 (40.6%)	32 (37.6%)
Age at diagnosis		
≤ 60	72 (40%)	34 (40%)
> 60	108 (60%)	51 (60%)
Cigarette smoking		
No	157 (87.2%)	63 (74.1%)
Yes	23 (12.8%)	22 (25.9%)
DM		
No	135 (75%)	62 (72.9%)
Yes	45 (25%)	23 (27.1%)
Pre‐CRT CEA (ng/mL)		
≤ 5	123 (68.3%)	35 (41.2%)
> 5	57 (31.7%)	50 (58.8%)
Distance from anal verge		
≤ 5 cm	70 (38.9%)	29 (34.1%)
5–10 cm	69 (38.3%)	28 (32.9%)
10–15 cm	33 (18.3%)	22 (25.9%)
Unknown	8 (4.4%)	6 (7.1%)
Clinical T stage		
T2	5 (2.8%)	2 (2.4%)
T3	150 (83.3%)	68 (80.0%)
T4	25 (13.9%)	15 (17.6%)
Clinical N stage		
N0	39 (21.7%)	15 (17.6%)
N+	141 (78.3%)	70 (82.4%)
RT to surgery interval		
> 8 weeks	169 (93.9%)	83 (97.6%)
≤ 8 weeks	11 (6.1%)	83 (2.4%)
Pre‐OP CEA (ng/mL)		
≤ 2	87 (48.3%)	30 (35.3%)
> 2	93 (51.7%)	55 (64.7%)

Abbreviations: CEA, carcinoembryonic antigen; CRT, chemoradiation therapy; DM, diabetes mellitus; Pre‐OP, preoperative; RT, radiotherapy; TRG, tumor regression grade.

### 3.2 Univariate and Multivariate Predictors

The univariate analysis revealed that smoking (*p* = 0.008), a pre‐CRT CEA level of > 5 ng/mL (*p* < 0.001), and a preoperative CEA level of > 2 ng/mL (*p* = 0.046) were significantly associated with a higher TRG (Table 2). Subsequently, all factors were included in the multivariate logistic regression, and only smoking and a higher pre‐CRT CEA level remained significant predictors of a higher TRG. The HRs were 2.32 (95% CI: 1.06–5.07, *p* = 0.036) for smoking and 3.1 (95% CI: 1.69–5.68, *p* < 0.001) for a higher pre‐CRT CEA level. Further analysis of different stages in non‐smokers and smokers revealed no significant difference in T or N stages between the two groups (*p* = 0.059 and *p* = 0.635, respectively) (Table S1). Additionally, a subsequent multivariate logistic regression analysis conducted within the non‐smoking group (*n* = 220) showed that a higher pre‐CRT CEA level remained a significant predictor of a higher TRG, with a hazard ratio (HR) of 3.01 (95% CI: 1.52–5.95, *p* < 0.002) (Table S2).

**TABLE 2:** Results of univariate and multivariate Cox regression for factors associated with TRG.

Variables	Univariate		Multivariate	
	Hazard ratio (95% CI)	*p*	Hazard ratio (95% CI)	*p*
Gender		0.651		0.971
Female	1.00		1.00	
Male	1.13 (0.67–1.92)		1.01 (0.54–1.91)	
Age at diagnosis		1.000		0.875
≤ 60	1.00		1.00	
> 60	1.00 (0.59–1.69)		1.05 (0.57–1.92)	
Cigarette smoking		0.008*		0.036*
No	1.00		1.00	
Yes	2.38 (1.24–4.58)		2.32 (1.06–5.07)	
DM		0.720		0.653
No	1.00		1.00	
Yes	1.11 (0.62–2.00)		0.86 (0.44–1.68)	
Pre‐CRT CEA (ng/mL)		< 0.001*		< 0.001*
≤ 5	1.00		1.00	
> 5	3.08 (1.81–5.26)		3.10 (1.69–5.68)	
Distance from anal verge		0.305		0.440
≤ 5 cm	1.00		1.00	
5–10 cm	0.98 (0.53–1.82)		0.86 (0.44–1.68)	
10–15 cm	1.61 (0.81–3.21)		1.42 (0.66–3.07)	
Clinical T stage		0.724		0.928
T2	1.00		1.00	
T3	1.13 (0.21–5.99)		1.15 (0.09–13.99)	
T4	1.50 (0.26–8.72)		1.34 (0.10–18.45)	
Clinical N stage		0.448		0.705
N0	1.00		1.00	
N+	1.29 (0.67–2.50)		0.86 (0.39–1.88)	
RT to surgery interval		0.186		0.225
> 8 weeks	1.00		1.00	
≤ 8 weeks	2.70 (0.59–12.47)		2.89 (0.47–17.92)	
Pre‐OP CEA (ng/mL)		0.046*		0.405
≤ 2	1.00		1.00	
> 2	1.72 (1.01–2.92)		1.31 (0.70–2.46)	

*Note*: *p < 0.05.

Abbreviations: CEA, carcinoembryonic antigen; CRT, chemoradiation therapy; DM, diabetes mellitus; Pre‐OP, preoperative; RT, radiotherapy; TRG, tumor regression grade.

### 3.3 Survival Analysis

**FIGURE 2:**
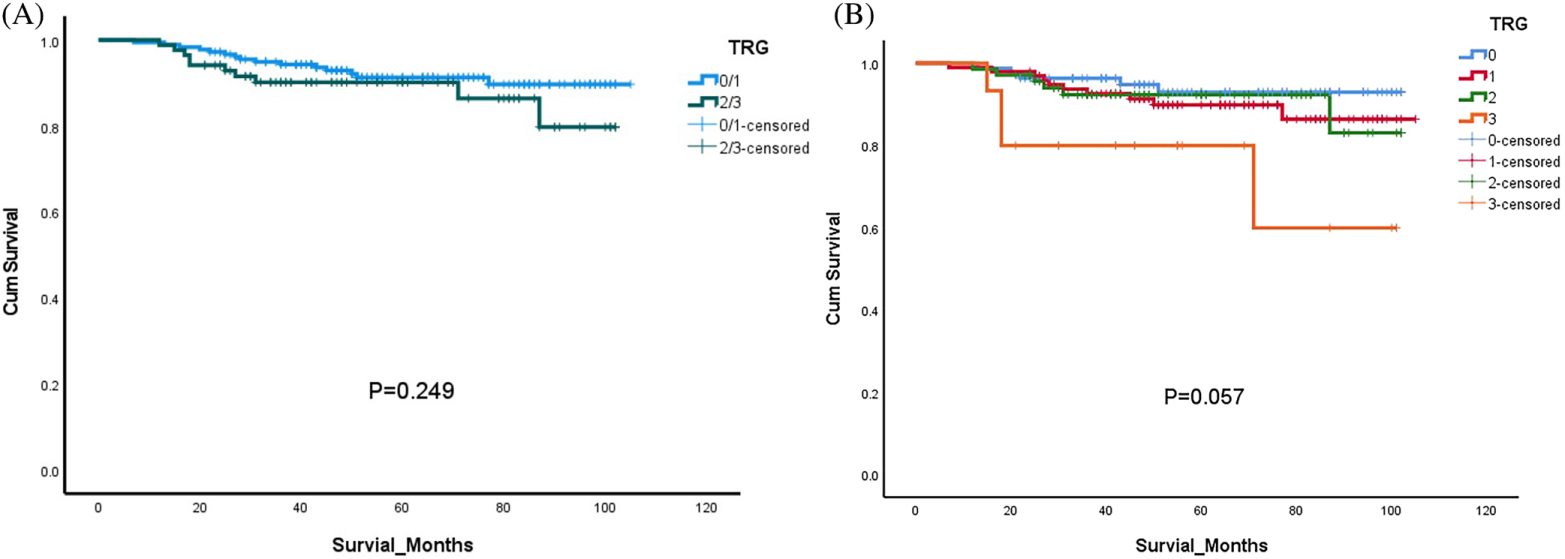
Overall survival differences among (A) two subgroups (TRG 0/1, TRG 2/3) (B) four subgroups (TRG0, TRG1, TRG2, TRG3).

**FIGURE 3:**
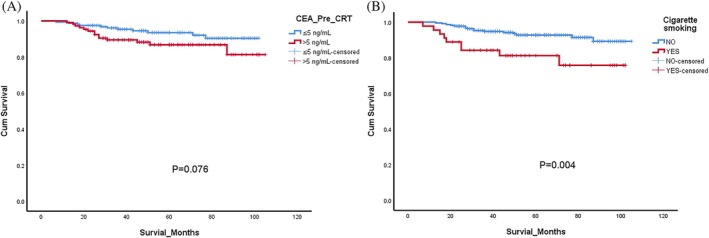
Overall survival of (A) two prechemoradiotherapy carcinoembryonic antigen (CEA_Pre_CRT) level groups and (B) smokers and nonsmokers.

In an unmatched analysis, the survival rate did not differ significantly among the two broad TRG subgroups, four TRG subgroups, and two pre‐CRT CEA subgroups (*p* values of 0.249, 0.057, and 0.076, respectively; Figures 2 and 3A). In a subsequent analysis stratified by smoking status, the nonsmoker group exhibited greater OS, as indicated by the results of Kaplan–Meier analysis (*p* = 0.004; Figure 3B). As indicated in Table 3, further analysis of the smoker subgroup revealed that in the nine patients who died, the primary causes of death were sepsis (*n* = 3; 33.3%) and cancer recurrence (*n* = 3; 33.3%). In the non‐smoker group, 16 patients died. Unlike the smoker group, the primary causes of death in the non‐smoker group were sepsis (*n* = 8; 50%) and respiratory failure (*n* = 5; 31%), while cancer recurrence accounted for only 6% (*n* = 1) (see Table S3).

**TABLE 3:** Deceased patients with LARC who smoked.

Age (year‐old)	Gender	TRG	Clinical stage	Cause of death
28	Male	2	3C	Cancer recurrence
52	Male	3	3C	Sepsis
61	Male	1	3C	Sepsis
61	Male	2	3B	Respiratory failure
63	Male	0	3C	Cancer recurrence
64	Male	3	3B	Cancer recurrence
64	Male	3	3B	Heart failure with preserved ejection fraction
82	Male	2	3B	Pneumonia
83	Male	1	2A	Sepsis

## 4 Discussion

### 4.1 AJCC TRG System

NACRT is widely accepted as the gold standard for treating LARC and is associated with improved survival outcomes and the preservation of organ function [4]. To determine TRG, the ratio of residual cancer cells to radiation‐induced fibrosis is assessed, which enables clinicians to evaluate the effectiveness of neoadjuvant treatment and adjust subsequent therapeutic strategies on the basis of their evaluation. TRG is commonly incorporated into pathologic reports and is a standardized method for evaluating tumor response. For the tumor regression grade system established by the AJCC and the College of American Pathologists, a 3‐point grading scale is used. This system has superior reproducibility to that of other systems [11], and the regression grade of the system is a predictive factor for patients with rectal cancer, which indicates that it is clinically relevant [15]. The AJCC TRG system should be used for prospective evaluations of NACRT outcomes in patients with rectal cancer. In our study, TRG 3 was significantly associated with the lowest OS rate. This finding is consistent with that of another study that involved 185 patients with LARC [11].

### 4.2 Smoking and TRG

Our study demonstrated that smoking and pre‐NACRT CEA levels greater than 5 ng/mL are independent prognostic factors for higher a TRG in patients with LARC treated with NACRT followed by total mesorectal excision. Even after other well‐established prognostic factors were adjusted for using multivariate analysis, the prognostic value of smoking and pre‐CRT CEA levels greater than 5 ng/mL remained significant. Smoking is a risk factor for many types of cancer [24]. Moreover, it is a risk factor for the development of colorectal adenomas [25] and CRC‐related mortality [26]. However, few studies have addressed the association of smoking and outcomes of NACRT in patients with rectal cancer [27]. To the best of our knowledge, this study is the first to reveal an association between smoking and response to NACRT in LARC treatment. Our findings indicate that smoking significantly reduces the efficacy of NACRT in patients with LARC. Smokers faced a twofold increase in the risk of poor tumor regression, which was exacerbated by lower OS following treatment for rectal cancer. Sepsis and cancer recurrence were the primary causes of death among the individuals who smoked. This finding is in line with those of other studies [28, 29], which have demonstrated a robust dose–response relationship between smoking and poorer survival outcomes among patients with stage I–III CRC. The adverse effect of smoking is mostly associated with non‐CRC events; however, an association is also present between current heavy smoking and CRC‐related survival [29]. Smoking considerably enhances the risk of CRC developing through the microsatellite instability pathway, which is characterized by microsatellite instability—high, CpG island methylator phenotype positivity, and *BRAF* mutation [30, 31]. Patients with LARC with mismatch‐repair deficiency are exceptionally sensitive to blockade of single‐agent programmed cell death‐1, which is a key mediator of immune suppression within the tumor microenvironment in NACRT [32]. However, the specific mechanisms through which smoking undermines the effectiveness of NACRT in the treatment of LARC remain uninvestigated.

### 4.3 Pre‐CRT CEA Levels

Normal serum CEA levels independently predict long‐term survival in patients with advanced CRC [33]. In our earlier study on LARC, post‐CRT CEA levels were the primary predictor of pathologic complete response, followed by the time between preoperative NACRT and surgery, chemotherapy regimen, clinical nodal stage, and tumor stage [34]. Pre‐CRT CEA and post‐CRT CEA levels have shown inconsistent results regarding their ability to predict TRG in LARC treatment [35]. A retrospective study involving 269 patients indicated that, excluding smoking as a factor, the pre‐CRT CEA level did not correlate with TRG (*p* = 0.503), whereas the post‐NACRT CEA level was significantly associated with TRG [36]. Additionally, another study found that a lower pre‐neutrophil‐lymphocyte ratio was likely linked to a higher TRG rate in LARC patients receiving NACRT, while neither smoking nor pre‐CRT CEA levels showed significant associations [37]. The current study revealed that only pre‐NACRT serum CEA levels significantly affected tumor regression; post‐NACRT CEA levels were not associated with tumor regression.

Wallin et al. proposed that a link was present between low pretreatment CEA levels and pathologic complete response in nonsmokers undergoing NACRT for LARC [38]. Smoking has been shown to influence serum CEA levels, with several studies reporting that smokers tend to have higher CEA levels compared to non‐smokers [39, 40]. In our study, we demonstrated that pre‐CRT CEA levels independently influenced TRG in patients with LARC following NACRT (Table S1). And whether worsen TRG caused by cigarette smoking through the mechanism of increased CEA levels needed to be further studied. Patients with LARC with pre‐CRT CEA levels of less than 5 ng/mL had a higher probability of OS than did those with CEA levels of more than 5 ng/mL after treatment. However, the difference was not statistically significant. This lack of significance may be attributable to the statistical power being insufficient to detect small survival advantages.

### 4.4 Strengths and Limitations

This study has several strengths, such as its single‐center design, which ensured standardized treatment and testing protocols. This minimized the risk of data variability and allowed for robust and conclusive findings. Furthermore, dedicated health‐care professionals administered the preoperative concurrent CRT and performed the surgery, which ensured rigorous quality control throughout the study, including in the data collection and analysis.

Our study also has limitations. First, it was conducted at a single center and had a small sample size, which may limit the generalizability of the findings to a larger population. Additionally, complete molecular pathology biomarker data were not available for all patients, which prevented us from conducting multivariate analyses for different pathology stages and adjusting for potential confounders such as microsatellite instability, estimated glomerular filtration rate, *KRAS* expression levels, and ERCC1 levels. These factors can influence NACRT outcomes and should be investigated in future studies to ensure a more comprehensive understanding of their effects.

## 5 Conclusions

Our study indicates that smoking may be a risk factor for OS in patients with LARC, as observed in the univariate analysis. It also demonstrated that elevated pre‐CRT CEA levels and smoking are associated with a more than twofold increase in the risk of poor tumor regression following neoadjuvant therapy in patients with LARC. Therefore, health‐care professionals must implement smoking cessation strategies for individuals who smoke and have rectal cancer. Furthermore, the underlying mechanisms linking smoking to a weaker response to NARCT in patients with LARC warrant further investigation.

## Conflicts of Interest

The authors declare no conflicts of interest.

## Supporting information


Table S1.
Table S2.
Table S3.

## Data Availability

The data that support the findings of this study are available from the corresponding author upon reasonable request.
