# Supplementary material for: Smoking and Elevated Preneoadjuvant Chemoradiotherapy Serum Carcinoembryonic Antigen Levels Are Associated With High Tumor Regression Grade and Poor Survival in Patients With Locally Advanced Rectal Cancer
Source: Kaohsiung J Med Sci. 2025 Mar 13;41(6):e70008. doi: 10.1002/kjm2.70008 (PMC12199573; doi:10.1002/kjm2.70008)
Supplement: Supplementary file 1 — Table S1. Table S2. Table S3. [file KJM2-41-e70008-s001.docx]

Supplement Table1

Analysis of different stages in non-cigarette smokers and cigarette smokers

|  | Non-Cigarette smokers  (n=220) | Cigarette smokers  (n=45) | *p-*value |
| --- | --- | --- | --- |
| Clinical T stage |  |  | 0.059 |
| T2 | 6 (2.7%) | 1 (2.2%) |  |
| T3 | 186 (84.5%) | 32 (71.1%) |  |
| T4 | 28 (12.7%) | 12 (26.7%) |  |
| Clinical N stage |  |  | 0.635 |
| N0 | 46 (20.9%) | 8 (17.8%) |  |
| N+ | 174 (79.1%) | 37 (82.2%) |  |

Supplement Table2

Results of multivariate Cox regression for factors associated with TRG in non-smoker patients.

| Variables | Multivariate | |
| --- | --- | --- |
|  | Hazard ratio  (95% CI) | *P*-value |
| Gender |  | 0.831 |
| Female | 1.00 |  |
| Male | 1.07 (0.56-2.06) |  |
| Age at diagnosis |  | 0.929 |
| ≤60 | 1.00 |  |
| >60 | 0.97 (0.49-1.92) |  |
| DM |  | 0.806 |
| No | 1.00 |  |
| Yes | 1.10 (0.52-2.31) |  |
| Pre-CRT CEA (ng/mL) |  | <0.002* |
| ≤5 | 1.00 |  |
| >5 | 3.01 (1.52-5.95) |  |
| Distance from anal verge |  | 0.323 |
| ≤ 5 cm | 1.00 |  |
| 5–10 cm | 0.84 (0.45-1.58) |  |
| 10-15 cm | 1.44 (0.67-3.07) |  |
| Clinical T stage |  | 0.340 |
| T2 | 1.00 |  |
| T3 | 1.25 (1.03-13.99) |  |
| T4 | 1.36 (0.25-8.45) |  |
| Clinical N stage |  | 0.219 |
| N0 | 1.00 |  |
| N+ | 0.59 (0.25-1.37) |  |
| RT to surgery interval |  | 0.924 |
| >8 weeks | 1.00 |  |
| ≤8 weeks | 1.09 (0.20-5.80) |  |
| Pre-OP CEA (ng/mL) |  | 0.181 |
| ≤2 | 1.00 |  |
| >2 | 0.62 (0.30-1.25) |  |

TRG: tumor regression grade; DM, diabetes mellitus; CRT, chemoradiation therapy; CEA, carcinoembryonic antigen; RT: radiotherapy; Pre-OP, preoperative.

Supplement Table 3. Deceased patients with LARC who non-smoked

| **Age (year-old)** | **Gender** | **TRG** | **Clinical stage** | **Cause of death** |
| --- | --- | --- | --- | --- |
| 46 | Female | 0 | 3B | Cancer recurrence |
| 52 | Female | 1 | 3C | Respiratory failure |
| 54 | Female | 1 | 3B | Sepsis |
| 56 | Male | 2 | 2A | Sepsis |
| 56 | Female | 1 | 2A | Respiratory failure |
| 57 | Female | 1 | 3B | Sepsis |
| 57 | Male | 2 | 3B | Respiratory failure |
| 60 | Male | 0 | 3B | hypovolemic shock |
| 61 | Male | 1 | 3B | Respiratory failure |
| 63 | Male | 0 | 3C | Sepsis |
| 64 | Female | 3 | 3B | Sepsis |
| 70 | Male | 2 | 3B | Septic |
| 80 | Male | 1 | 3B | UTI, cachexia |
| 81 | Female | 0 | 3B | Respiratory failure |
| 88 | Female | 1 | 3B | Sepsis |
| 90 | Male | 1 | 2A | Sepsis |
